# The mechanism of solvent-mediated desolvation transformation of lenvatinib mesylate from di­methyl sulfoxide solvate to form D

**DOI:** 10.1107/S2052520620003935

**Published:** 2020-05-07

**Authors:** Zhixin Zheng, Baohong Hou, Xiaowei Cheng, Wanying Liu, Xin Huang, Ying Bao, Ting Wang, Zhao Wang, Hongxun Hao

**Affiliations:** a National Engineering Research Center of Industrial Crystallization Technology, School of Chemical Engineering and Technology, Tianjin University, Tianjin, 300072, People’s Republic of China; b Collaborative Innovation Center of Chemical Science and Engineering (Tianjin), Tianjin, 300072, People’s Republic of China

**Keywords:** lenvatinib mesylate, solubility, solvent-mediated desolvation transformation, water activity, mechanism

## Abstract

In this work, the mechanism of solvent-mediated desolvation transformation of lenvatinib mesylate (LM) was investigated. Two new solid forms of LM, a di­methyl sulfoxide (DMSO) solvate and an unsolvated form defined as form D, were discovered and characterized using powder X-ray diffraction, thermogravimetric analysis, differential scanning calorimetry, polarized light microscopy and Raman spectroscopy. To investigate the thermodynamic mechanism of solvent-mediated desolvation transformation (SMDT) from LM DMSO solvate to form D, solubilities of LM DMSO solvate and form D in binary solvent mixtures of DMSO and water at different water volume fractions and temperatures (293.15–323.15 K) were measured and correlated by non-random two liquids model. The solubility data were used to evaluate the thermodynamic driving force of the SMDT process from DMSO solvate to form D and the effect of the activities of water and DMSO on the transformation process. Raman spectroscopy was used to monitor *in situ* the solid phase compositions during the SMDT process from LM DMSO solvate to form D while the solution concentration was measured by the gravimetric method. The overall desolvation transformation experiments demonstrated that the SMDT process was controlled by the nucleation and growth of form D. Moreover, effects of operating factors on the SMDT process were studied and the results illustrated that water activity in solution was the paramount parameter in the SMDT process. Finally, a new SMDT mechanism was suggested and discussed.

## Abstract 2

The solvent-mediated desolvation process of newly discovered lenvatinib DMSO solvate to form II at different water volume fractions and temperatures was investigated. It is confirmed that the activity of water is the most important factor affecting the desolvation process: the desolvation process only occurs when the activity of water is greater than the activity of DMSO, and one new mechanism of solvent-mediated desolvation process was proposed.

## 1. Introduction   

Nowadays, screening new solid forms of an active pharmaceutical ingredient (API) is an indispensable step in the development and production of drugs. The physical properties of different solid forms of the same compound, such as melting point, solubility, dissolution rate, physical and chemical stability, *etc*., may vary significantly. These properties will further affect the bioavailability, fluidity and efficacy of the final products (Schöll *et al.*, 2006 ▸; Llinàs & Goodman, 2008 ▸; Bērziņš *et al.*, 2017 ▸; Brittain, 2009 ▸; Bond, 2009 ▸). Different crystal forms of multifarious drugs have been screened by controlling various factors influencing the crystallization process, such as temperature, solvent, humidity, solid loading, stirring rate, additives and template materials (Jiang *et al.*, 2015 ▸; Hao *et al.*, 2010 ▸, 2012 ▸; Guo *et al.*, 2018 ▸; Barbas *et al.*, 2018 ▸; Shi *et al.*, 2015 ▸; Zong *et al.*, 2017 ▸; Yang *et al.*, 2013 ▸, 2012 ▸; Ouyang *et al.*, 2014 ▸). In fact, many substances have far more solvated forms than the unsolvated form. For example, methyl cholate has eight unsolvated forms and 27 solvates (Bērziņš *et al.*, 2017 ▸). Sulfa­thia­zole has more than 100 solvates but only five unsolvated forms (Anwar *et al.*, 1989 ▸; Apperley *et al.*, 1999 ▸; Bingham *et al.*, 2001 ▸). Therefore, the study of solvates should be of great concern.

Solvates have a wide application prospect. Firstly, solvates can be used in the purification of substances. For example, form R_1_ of S-enzalutamide can be easily contaminated by its substitution impurity O-enzalutamide. However, the impurity can be removed by desolvating iso­propanol solvate R_3_ which is formed by the solvation of form R_1_ (Maini *et al.*, 2018 ▸). Dirithromycin and piper­idene can also be purified from erythromycyl­amine and the ‘N linked’ impurity precursor of piper­idene by their acetone solvates and hydrates, respectively (Wirth & Stephenson, 1997 ▸; Black *et al.*, 2004 ▸). Secondly, solvates having superior properties can be used in the form of a commercial product, such as the DMSO solvate of trametinib, the acetone solvates of cabazitaxel and ledipasvir, the ethyl acetate solvate of voxilaprevir, *etc*. Thirdly, new polymorphs can be obtained by desolvating specific types of solvates. For instance, form D of prilocaine hydro­chloride can only be prepared by desolvating its dioxane solvate (Schmidt *et al.*, 2004 ▸) and its new crystal form AH D can only be obtained by the dehydration of thymine hydrate (Braun *et al.*, 2016 ▸). Therefore, it is necessary to carry out intensive investigations on the solvate formation and desolvation process.

Solvent-mediated solid phase transformation is one kind of transformation process from a metastable solid form to a more stable solid form in a solvent environment (Mangin *et al.*, 2009 ▸), which includes the dissolution of metastable solid form and the nucleation and growth of a stable solid form (Gu *et al.*, 2001 ▸; Jiang *et al.*, 2010 ▸). The determination of the rate control step of the solvent-mediated solid phase transformation process is essential to explore the transformation mechanism and better control of the transformation process. The rate control steps in the transformation process are different for different APIs and solvent systems. According to the real-time relationship between the completion degree of solid phase and the concentration of solution in the solid phase transformation process, O’Mahony *et al.* (2012 ▸) summarized four diverse circumstances, namely dissolution-controlled, growth-controlled, nucleation–dissolution-controlled and nucleation-growth-controlled solid phase transformation processes.

**Figure 1:**
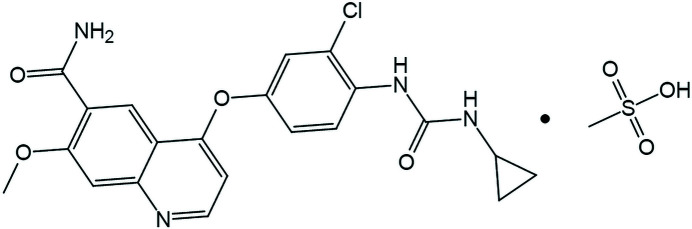
The molecular formula of lenvatinib mesylate, where the moculare formila on the left is molecular lenvatinib and the molecule on the right is a methansulfonic acid molecule.

The model compound lenvatinib mesylate (CAS registry NO: 857890-39-2, C_22_H_23_ClN_4_O_7_S; the chemical structure is shown in Fig. 1 ▸), 4-[3-chloro-4-(*N*′-cyclo­propyl­ureido)phen­oxy]-7-meth­oxy­quinoline-6-carboxamide methane sulfonate (denoted as LM), is one kind of oral multikinase inhibitor. Being a great-promising anticancer drug, LM has passed phase 3 clinical trials for differentiated thyroid cancer and is undergoing phase 3 clinical trials for liver cancer (Okamoto *et al.*, 2015 ▸). Until now, five unsolvated crystal forms, three hydrate crystal forms, two acetic acid solvates, one chloro­form solvate, and one formic acid solvate of LM have been discovered (Matsushima *et al.*, 2005 ▸; Sardone *et al.*, 2018 ▸). All the discovered solid forms of lenvatinib mesylate are listed in Table 1 ▸. However, the reported forms are more or less flawed. Form B will slowly transform to form C under high humidity conditions while the solubility of stable form C is too low and hydrate form F will transform to form C in a solution system with a water activity ranging from 0 to 0.821. Acetic acid solvate form I is not only hygroscopic but also unstable. Because of chloro­form belonging to the second class of solvents which are restricted (FDA classification of solvents) in use and formic acid is strongly acidic, chloro­form solvate CHF-1 and formic acid solvate FOA-1 are not applicable for pharmaceutical purposes. Acetic acid solvate ACA-1, anhydride ACA-1 HT dry and H_2_O-1 are all thermodynamically unstable. Moreover, the transformation relationships of different forms were not well investigated or understood. Thus, it is necessary to further investigate the solid state forms, crystal structures and crystal transformation behaviors of LM, which is essential for pharmaceutical development.

In this study, a new DMSO solvate and a new unsolvated form (form D) of LM were discovered and characterized for the first time. The thermodynamic mechanism of the solvent-mediated desolvation transformation (SMDT) process from DMSO solvate to form D in DMSO–water mixed solvent was evaluated by measuring and analyzing their solubility data. The SMDT process was investigated in detail. The composition of the solid phase was *in situ* monitored using Raman spectroscopy and the results were verified by quantitative powder X-ray diffraction. At the same time, the change of solute concentration in solution over time was determined through gravimetric analysis. Finally, the impacts of water activity and temperature on the SMDT process were investigated and discussed. A new SMDT mechanism was proposed based on the obtained results.

## 2. Experimental   

### 2.1. Materials   

The raw material of LM (form C) with a mass fraction purity higher than 99.0% was purchased from Jinan Sanzhi Pharmaceutical Technology Co., Ltd. DMSO and aceto­nitrile which are of analytical grade were supplied from Tianjin Kemiou Chemical Reagent Co., Ltd. Distilled water was prepared in our laboratory and used throughout all experiments.

### 2.2. Preparation of two solid forms of LM   

LM DMSO solvate was prepared from LM form C through a cooling-antisolvent coupled crystallization method. LM form C (1.00 g) was dissolved in DMSO (15 ml) under the stirring rate of 300 rpm (controlled by Vertical Constant Speed Electric Mixer, HD2015W, Shanghai Sile Instrument Co., Ltd) at 343.15 K (controlled by Thermostatic Water Bath, XOYS-2006N, Nanjing Xianou Instruments Manufacture Co., Ltd and measured by a mercury thermometer). After the solid was completely dissolved, aceto­nitrile (50 ml) was added into the solution at a rate of 0.1 ml min^−1^ by a peristaltic pump (Dispensing Peristaltic Pump, BT100-1F, Baoding Longer Precision Pump Co., Ltd) while the temperature was decreased to 293.15 K at a rate of 0.1 K min^−1^ and then maintained at constant temperature of 293.15 K for 8 h. Finally, the suspension was filtered and dried under vacuum at 313.15 K (Electrothermal Constant Temperature Drying Oven, DZ-2BCII, Tianjin Taisite Instrument Co., Ltd) to obtain LM DMSO solvate product.

LM form D was prepared from DMSO solvate by suspension transformation method. LM DMSO solvate (1 g) was added to a mixture of DMSO (15 ml) and water (5 ml) and stirred at 293.15 K with a stirring rate of 300 rpm for 24 h. After the transformation process was terminated, the suspension was filtered and dried under vacuum at 313.15 K to obtain LM form D product.

### 2.3. Characterization methods   

Powder X-ray diffraction (PXRD) patterns of LM DMSO solvate and form D were obtained using a Rigaku D/max-2500 diffractometer in 2θ range from 2° to 40° with a scanning rate of 8° min^−1^, step size of 0.08° and voltage of 40 kV and current of 100 mA.

A polarized light microscope (Series Biological Microscope, BK5000, Chongqing Optec Instrument Co., Ltd) was used to observe the exterior morphology of LM DMSO solvate and form D.

Thermogravimetric analysis (TGA) on a Mettler Toledo TASDT-Q600 instrument was used to determine the weight loss of LM DMSO solvate and form D. The temperature range was from 303.15 to 623.15 K with a heating rate of 5 K min^−1^ under a nitrogen atmosphere and the sample weight of 5–10 mg.

Differential scanning calorimetry (DSC) on a Mettler-Toledo DSC1 instrument was used to monitor the melting processes of LM DMSO solvate and form D. The samples of 5–10 mg were loaded into an aluminium crucible and scanned in the temperature range from 303.15 to 623.15 K with a heating rate of 5 K min^−1^ in a nitrogen atmosphere, so as to obtain the melting point and monitor the melting process of the two forms.

Raman spectra were collected with a Kaiser Raman RXN2 system (Kaiser Optical System, Inc., Ann Arbor, MI, USA). Solid powders were analyzed with a PhAT probe which was perpendicularly fixed above the solid powders at a distance of 2–3 mm. In transformation experiments, the immersion MR probe was inserted into the crystallizer and immersed into the suspension. Tin foil was used for shading during the whole experiment process. Raman spectra were collected in the range 100 cm^−1^ to 3200 cm^−1^ with a resolution of 0.5 cm^−1^ and an exposure time of 15 s.

### 2.4. The establishment of quantitative analysis method   

In order to confirm the mass fraction of the two crystal forms in the solid mixture, a PXRD calibration curve was established (Shi *et al.*, 2018 ▸). It is well known that the intensity of the PXRD diffraction peaks of a crystal can be affected by a variety of factors, such as powder packing, sample thickness, crystallite size, type of sample holder and preferred orientation effects of the crystal (Li *et al.*, 2011 ▸; Qiu *et al.*, 2015 ▸; Croker *et al.*, 2012 ▸). These factors were all taken into account when measuring the diffraction pattern of standard samples to minimize their influence on the result.

The standard samples were pure DMSO solvate, pure form D and mixtures of two forms with different proportions (mass fraction of DMSO solvate from 0.1 to 0.9, step size of 0.1). DMSO solvate and form D powder samples were ground individually in an agate mortar for 5 min and screened through a 400-mesh sieve to reduce the effect of crystal size on the preferred orientation. The standard samples were obtained by mixing the above powders of two forms in different proportions. Moreover, the sample stage and sample thickness were maintained at the same conditions to eliminate their influences on the final results during the PXRD diffraction pattern measurements. Meanwhile, all the standard samples were scanned from 2° to 20° on the 2θ scale with a scanning speed of 8° min^−1^ and a step size of 0.02°.

In the quantitative process, 2θ = 6.9 ± 0.2° was selected as the characteristic peak of DMSO solvate while 2θ = 12.3 ± 0.2° was selected as the characteristic peak of form D. Since the intensity of the characteristic peak of DMSO solvate and form D are approximately equal under the same test conditions, the relative characteristic peak intensity of DMSO solvate can be selected to measure the mass fraction of DMSO solvate in the mixture, which can be calculated by equation (1):
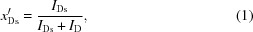
where *x*′_Ds_ is the calculated value of the mass fraction of DMSO solvate in the mixture, *I*_Ds_ and *I*_D_ represent the characteristic peak intensity of DMSO solvate and form D, respectively.

### 2.5. Solubility measurements and transformation equilibrium of two solid forms of LM   

Solubility data of LM DMSO solvate and form D in mixed solvents of DMSO and water were measured from 293.15 to 323.15 K. It was found that when the volume fraction of water in DMSO–water mixed solvent (denoted as *V*_w_) is higher than 0.300, the viscosity of the system increased rapidly, resulting in the inability to obtain a clear saturated solution. Thus, only the solubility data with *V*_w_ ranging from 0 to 0.300 and a step of 0.025 was obtained. *V*_w_ = 0.250 was taken as an example to explain the solubility measurement process: Excess LM form D was dissolved in a mixed solvent of 15 ml DMSO and 5 ml water to saturate the solution. The suspension was stirred at a stirring rate of 300 rpm for 24 h and the temperature was controlled by a thermostatic water bath. Then, the suspension was filtered and the PXRD of the undissolved crystals was measured to identify the undissolved crystals as form D, which proved that no crystal transformation occurred during the dissolution process. A clean beaker was weighed by an electronic balance (Analytical Balance, AL104, Mettler-Toledo) and the mass of it was recorded as *m*_0_. The filtrate was filtered through a syringe filter (0.22 µm) (Tianjin Legg Technology Co., Ltd, Tianjin, China) into the beaker and the total mass of the beaker and the filtrate was weighed again and recorded as *m*_1_. The filtrate was dried under vacuum at 373.15 K and weighed at intervals. When the mass remained unchanged twice, the solvent could be considered to be completely evaporated and the final mass was recorded as *m*_2_. The molar solubility of form D can be calculated by equation (2). Each experiment was performed three times to eliminate the error, and the final solubility was taken as the average of the three experimental results.
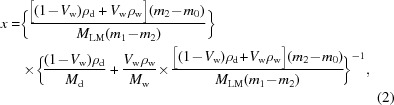
where *x* is the molar fraction solubility; ρ_d_ and ρ_w_ represent the density of DMSO and water at specific temperature respectively, with unit of g ml^−1^; *M*_d_, *M*_w_, and *M*_LM_ are the molar masses of DMSO, water and LM respectively, with unit of g ml^−1^; *V*_w_ is the volume fraction of water in DMSO–water mixed solvent. The values of parameters in equation (2) are shown in Table 2 ▸.

**Figure 2:**
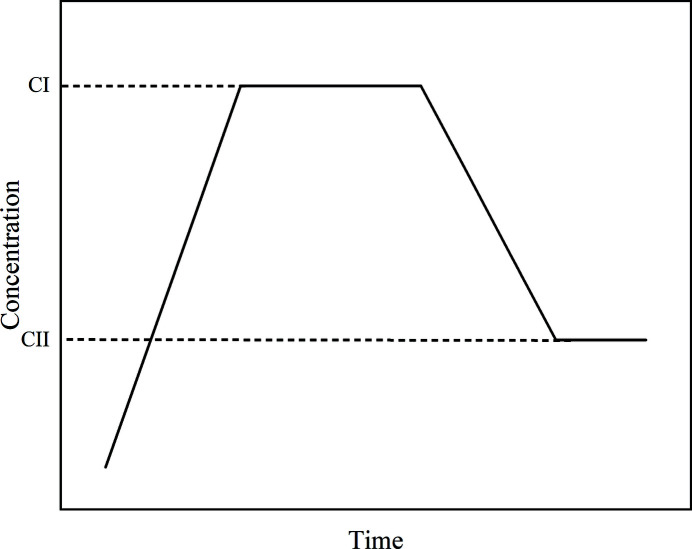
Schematic diagram of the concentration of solution as a function of time, which displays the process of transformation from metastable form to stable form. CI is the solubility of the metastable form and CII is the solubility of the stable form.

Since DMSO solvate will transform to form D, it is difficult to measure its solubility by conventional gravimetric methods. In the process of solvent-mediated polymorphic transformation (SMPT) from metastable form to stable form, the concentration of the solution will undergo three stages of change (Du *et al.*, 2014 ▸), as shown in Fig. 2 ▸. During the dissolution stage of the metastable form, the solution concentration will rise from zero to the solubility of the metastable form and remain constant. When the stable form starts to nucleate and grow, the solute consumed by the nucleation and growth of stable form will be balanced by the dissolution of the metastable form. Thus, the concentration of the solution will be maintained at the solubility of the metastable form if the crystallization process is controlled by the nucleation and growth of the stable form. However, the concentration of the solution will drop to the solubility of the stable form and remain constant when the metastable form in solution is completely dissolved or the process of crystal transformation is controlled by the dissolution of the metastable form.

The solubility of LM DMSO solvate was measured by using real time Raman spectroscopy. Excess DMSO solvate was dissolved in a mixed solvent of DMSO (15 ml) and water (5 ml) to saturate the solution, while the temperature was controlled by a thermostatic water bath. Raman spectroscopy was used to collect *in situ* the Raman spectra of the solution in one minute intervals. When the characteristic peak intensity of DMSO solvate decreased, the experiment was immediately stopped. The suspension was filtered to obtain undissolved crystals and the PXRD data were measured to identify the undissolved crystals. The filtrate was filtered through a syringe filter and dried under vacuum at 373.15 K. The solubility of DMSO solvate can be calculated by the equation (2).

The non-random two liquids (NRTL) equation is an activity coefficient model based on the concept of local composition and it can be used to calculate the activity of substances in the system. The NRTL equation in the ternary system can be simplified as:
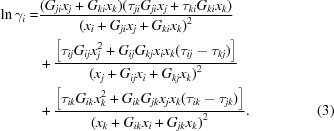

*G*_*ij*_ and τ_*ij*_ can be calculated by equations (4) and (5).
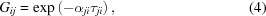


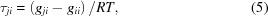
where *g*_*ji*_ − *g*_*ii*_ is the basic parameter of the NRTL equation which denotes the cross interaction energy. The parameter α_*ji*_ is related to the non-randomness in the mixture. The value of α_*ji*_ must be greater than 0 and less than 1. Parameter α_*ji*_ in the ternary system follows equation (6)

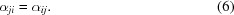



### 2.6. Real-time monitoring of the SMDT process from LM DMSO solvate to form D using Raman spectroscopy   

The SMDT process from LM DMSO solvate to form D was *in situ* monitored using Raman spectroscopy and the corresponding rate control step was ascertained. Moreover, the influences of water activity and temperature on the transformation process were also investigated. The effect of water activity on the SMDT process was investigated at 293.15 K, in which *V*_w_ varied between 0 and 0.300 with a step size of 0.025 because of the impossibility of obtaining products when *V*_w_ is higher than 0.300. The effect of temperature on the SMDT process was conducted at *V*_w_ = 0.250 in the temperature range of 293.15–323.15 K with the step of 10 K. At each temperature and *V*_w_, DMSO solvate (2.0 g) was added to DMSO–water mixed solvent (3:1 *v*/*v*, 40 ml) in a 100 ml crystallizer and the stirring rate was controlled to be 300 rpm with an overhead agitator. In all experiments, the systems were *in situ* monitored by Raman spectroscopy while the concentration of the solution was measured at certain intervals. The induction time was defined as the time from the starting of the experiment to the sudden drop of the Raman characteristic peak intensity of DMSO solvate, while the transformation time was defined as the time when the Raman characteristic peak intensity of DMSO solvate dropped to a constant value.

## 3. Results and discussion   

### 3.1. Characterization of DMSO solvate and form D of LM   

The PXRD diffraction patterns of LM form A, form C, form DMSO-2, DMSO solvate and form D are shown in supporting information (Fig. S1). The PXRD diffraction patterns of other reported solid forms of LM (Matsushima *et al.*, 2005 ▸; Sardone *et al.*, 2018 ▸) are listed in Fig. S2. It can be found that the PXRD data of DMSO solvate and form D are different from reported forms of LM. DMSO solvate has characteristic peaks at 2θ = 6.9 ± , 9.0 ± , 13.2 ± , 19.5 ± , 24.0 ± , 25.6 ± , 27.2 ± , 31.6 ± 0.2° while form D has characteristic peaks at 2θ = 4.1 ± 0.2°, 8.1 ± 0.2°, 10.2 ± 0.2°, 12.3 ± 0.2°, 16.5 ± 0.2°, 26.7 ±0.2 °, 29.0 ± 0.2°.

Polarized light microscopy (PLM) was used to observe the morphology of DMSO solvate and form D of LM and the results are shown in supporting information (Fig. S3). DMSO solvate is in the shape of hexagonal flake while form D is needlelike. The TGA thermogram of DMSO solvate (Fig. S4) shows 12.71% weight loss at 433.15 K, which is consistent with the theoretical DMSO content in DMSO solvate of LM with a stoichiometric ratio of 1:1 (about 13.00%). form D shows no lose weight before decomposition, proving that it is an unsolvated form. The DSC curve of DMSO solvate (Fig. S5) shows an endothermic peak at 433.15 K, which corresponds to the position of weight loss in the TGA curve, indicating that it is a desolvation peak. Another endothermic peak at 503.15 K indicates that crystals formed after desolvation of DMSO solvate melts. The DSC curve of form D shows an endothermic peak at 473.15 K, which should be the melting temperature of form D.

Raman spectroscopy can be used to identify different polymorphs and can manifest the differences of their structures (Elbagerma *et al.*, 2010 ▸). The Raman spectra results of DMSO solvate and form D of LM are shown in Fig. S6. Peaks at 1395 cm^−1^ and 1373 cm^−1^ were selected as characteristic peaks for DMSO solvate and form D, respectively and these peaks will be used to characterize their crystal transformation behavior in this work.

### 3.2. Solubility and thermodynamic driving force for SMDT process   

**Figure 3:**
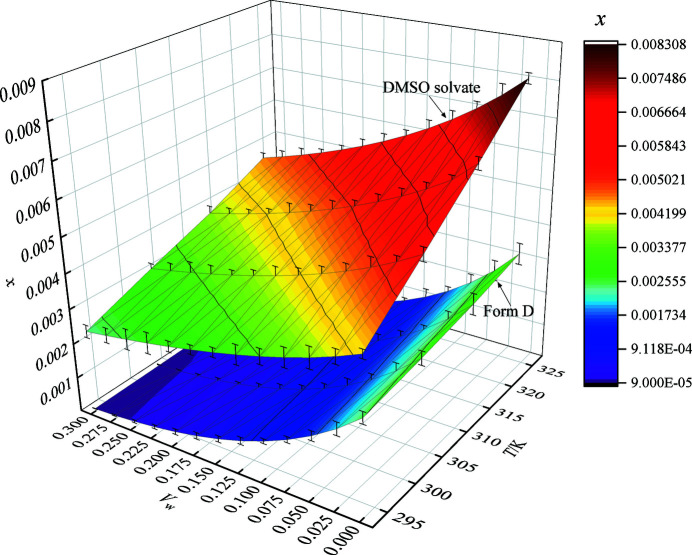
Solubility data of DMSO solvate and form D of LM in DMSO–water mixed solutions.

In the crystal transformation process, solubility data can be used to evaluate the relative stability of different forms and to investigate the thermodynamic mechanism of the transformation (Zong *et al.*, 2017 ▸; Du *et al.*, 2014 ▸; Jouyban, 2008 ▸). In this study, the solubility data of LM DMSO solvate and form D in the mixed solvents of DMSO and water were measured, and the results are shown in Fig. 3 ▸. Specific data are given in Table S1.

It can be comprehended from Fig. 3 ▸ that the solubility of LM DMSO solvate and form D increased with the decreasing of *V*_w_ and increasing of temperature. The solubility surfaces of DMSO solvate and form D do not intersect and the solubility surface of DMSO solvate is permanently above the solubility surface of form D under the investigated conditions, indicating that the solubility of DMSO solvate is always higher than that of form D under the investigated conditions. According to the solubility data, form D is the stable form in the investigated temperature range, which implies that DMSO solvate should have a tendency to transform to form D in DMSO–water mixed solvent, regardless of the volume fraction of water. Furthermore, the NRTL equation was used to calculate the activities of DMSO solvate/form D, water and DMSO in saturated solution systems. The results of activity are listed in Table S1.

The thermodynamic driving force of the SMDT process from DMSO solvate to form D can be described by the Gibbs free energy difference (Δ*G*_Ds→D_) of DMSO solvate and form D, which can be calculated by equation (7):
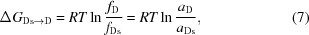
where *R* is the ideal gas constant, 8.314 J mol^−1^ K^−1^; *T* is the temperature with unit of K, *f* is the fugacity in units of Pa and *a* is the activity.

**Figure 4:**
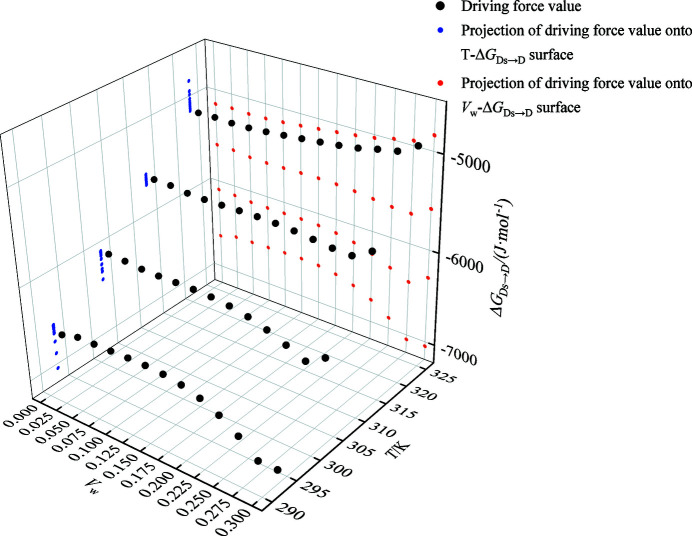
The thermodynamic driving force of SMDT from DMSO solvate to form D.

The relationship among the driving force of the SMDT process, the temperature and *V*_w_ is displayed in a 3D map (Fig. 4 ▸). The values of Δ*G*_Ds→D_ fluctuate within a smaller range with the increase of *V*_w_, indicating that the change of *V*_w_ have little effect on thermodynamic driving forces. In the temperature range of 293.15–323.15 K, the absolute values of Δ*G*_Ds→D_ decrease with the increase of temperature, indicating that the driving force of the SMDT process diminishes as temperature increases. All values of Δ*G*_Ds→D_ are negative, indicating that the SMDT process from DMSO solvate to form D should be spontaneous under the investigated conditions.

### 3.3. SMDT process *in situ* monitored using Raman spectroscopy and determination of rate-controlling step   

**Figure 5:**
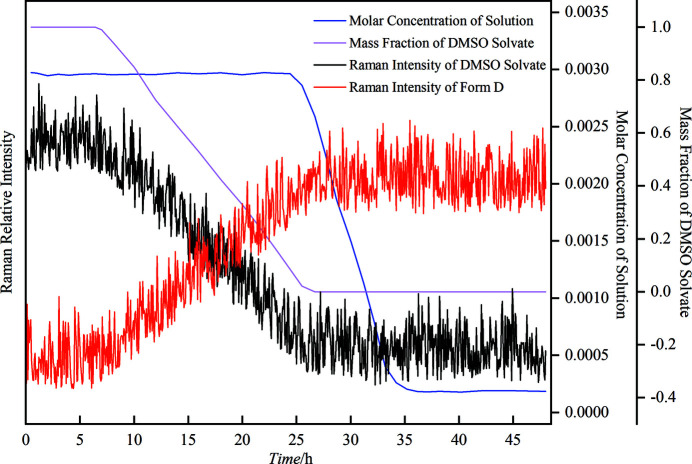
The SMDT process from DMSO solvate to form D in mixed solvent of DMSO and water (*V*_w_ = 0.250) at 293.15 K.

Raman spectroscopy was used to monitor *in situ* the SMDT process from LM DMSO solvate to form D. The suspension was taken out at fixed intervals during the SMDT process to analyze the liquid concentration and solid phase composition by gravimetric method and PXRD calibration curve, respectively. The representative results at *T* = 293.15 K and *V*_w_ = 0.250 are graphically shown in Fig. 5 ▸ and part of the PXRD patterns are shown in Fig. S8.

It can be seen from Fig. 5 ▸ that the trend of the mass fraction of DMSO solvate in the suspended solid phase measured by PXRD data coincide with the trend represented by the relative Raman intensity of the characteristic peak of DMSO solvate (1395 cm^−1^), manifesting that the real time Raman data could accurately represent the solid state composition of the SMDT process. Fig. S8 shows the change of suspended solid phase over time more intuitively. During the first 6.4 h of transformation process, the relative Raman intensity of the characteristic peak of DMSO solvate kept constant, which indicated that no transformation phenomenon occurred. The characteristic peak of form D (1373 cm^−1^) emerged at about 6.4 h, indicating the end of the induction process and the beginning of the nucleation and growth process of form D. Then, the relative Raman intensity of form D gradually increased while the relative Raman intensity of DMSO solvate decreased, revealing the proceeding of the transformation process. At approximately 26.7 h, the characteristic peak of DMSO solvate thoroughly vanished and the relative Raman intensity of form D reached maximum and remained unchanged, indicating that DMSO solvate had entirely transformed into form D, corresponding to the end of the SMDT process. Moreover, the liquid phase concentration curve shows that the solution concentration stayed at a plateau for 2.3 h before the transformation process was finished, indicating that the consuming rate of supersaturation by the growth of stable form was lower than the generating rate of supersaturation by the dissolution of metastable form (O’Mahony *et al.*, 2012 ▸). Then, the concentration gradually decayed to the saturation concentration of form D within about 11.8 h, and whereafter, remained unchanged. Moreover, the system underwent a long induction time (about 6.4 h) before the transformation process started, which may be caused by the comparative difficulty to form the crystal nuclei of stable form D. According to the four instances described by O’Mahony *et al.* (2012 ▸), the SMDT process from LM DMSO solvate to form D is controlled by the nucleation and growth of form D.

### 3.4. Effect of water activity on the SMDT process   

**Figure 6:**
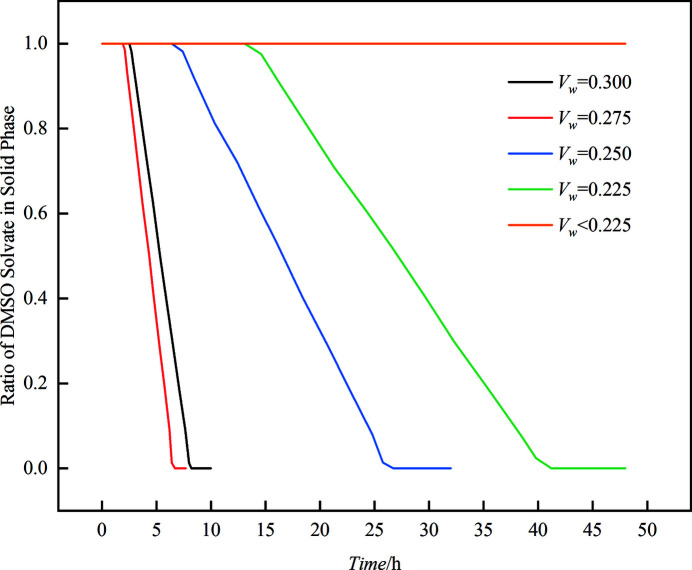
Effect of *V*_w_ on the SMDT process from LM DMSO solvate to form D at 293.15 K.

In this work, the effect of water activity on the SMDT process in the DMSO–water mixed solvent was investigated and the results are shown in Fig. 6 ▸ and Table 3 ▸.

It can be seen from Fig. 6 ▸ that the solvent molecules in DMSO solvate could be removed only when *V*_w_ is higher than or equal to 0.225. However, it is inconsistent with the conclusion from the thermodynamic driving force discussed in Section 3.2 that the SMDT process is spontaneous regardless of the *V*_w_. The contradiction demonstrates that the SMDT process probably follows a special mechanism which is not only controlled by the thermodynamic driving force. The activity of water might play a vital role in this process. It can be deduced from the experimental phenomenon and activity data that the necessary condition for the SMDT process is that the activity of water must be higher than the activity of DMSO. Based on these data, a new mechanism for the SMDT process of LM is proposed.

**Figure 7:**
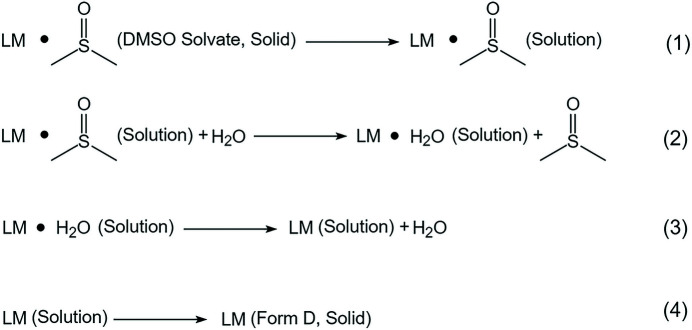
The mechanism of the SMDT process from LM DMSO solvate to form D.

The SMDT process from LM DMSO solvate to form D may be divided into four steps. Since the specific structure of lenvatinib mesylate was not successfully determined, the term ‘LM’ is used here to refer to the co-crystal/salt structure formed by one molecule of lenvatinib and one molecule of methane­sulfonic acid. Firstly, when DMSO solvate is dissolved in the mixed solvent, the intermolecular interaction (probably hydrogen bond) between LMs and DMSO molecules will not be broken straightaway. In other words, the structure of LM·DMSO could be maintained in the solution for some time. Secondly, water molecules might be promoted to replace DMSO molecules in the original structure after a period of induction time if the activity of water is higher than the activity of DMSO, which would lead to the formation of intermediates combined by the intermolecular interactions (probably by hydrogen bonds) of LMs and water molecules. Thirdly, water molecules in the intermediate molecular structure could also be removed, resulting in the freeing of LMs. Finally, isolated LMs might form new structures by solute-solute intermolecular interactions, which can lead to the start of the nucleation process of form D. The whole SMDT mechanism is demonstrated in Fig. 7 ▸. Overall, water might play the role of catalyst throughout the desolvation process. Thus, kinetically, when the activity of water is lower than the activity of DMSO, the impossibility of triggering the second step will inhibit the SMDT process, even if the desolvation of DMSO solvate is a thermodynamic spontaneous process.

In order to verify this mechanism, an extra cooling crystallization experiment was designed. Appropriate amount of LM DMSO solvate was added to a DMSO–water mixed solvent with *V*_w_ = 0.250 at 323.15 K to form a saturated solution. The saturated solution was cooled to 275.15 K at a cooling rate of 0.5 K min^−1^, and crystals were found to precipitate. After the cooling was completed, stirring was continued for 12 h at the constant temperature. After filtering and vacuum drying, the crystal product was taken for XRD characterization and it was found that the hydrated crystalline form DMSO-2 was obtained (Sardone *et al.*, 2018 ▸). TGA and DSC curve (shown in Figs. S10 and S11) verified that DMSO-2 is a tetrahydrate (the weight loss detected by TGA is 12.07%) and its desolvation temperature is about 379.25 K. The results of this cooling crystallization are consistent with the SMDT mechanism mentioned above. The rapid decreasing of temperature would promptly elevate the supersaturation of the system and significantly reduce the nucleation time, which would make LM·H_2_O have no enough time to remove water molecules from the structure. Generally, lower temperature will favor the formation of intermolecular hydrogen bonds (Kim *et al.*, 2020 ▸). Consequently, the tetrahydrate crystal form DMSO-2 was formed. Moreover, the desolvation temperature of DMSO-2 (about 379.25 K) is higher than the boiling point of water (373.15 K), revealing that water molecules participate in the formation of the lattice structure and were tightly bonded with LM through intermolecular interactions (Fátima Pina *et al.*, 2012 ▸; Chen *et al.*, 2008 ▸). Moreover, the desolvation energy of DMSO-2 is 10.2 kJ mol^−1^ according to the result of DSC, which is within the energy range of the corresponding intermolecular hydrogen bond (5–40 kJ mol^−1^) (Pauling, 1960 ▸), revealing that the intermolecular interaction in DMSO-2 is hydrogen bond. Therefore, the thermal analysis results are also consistent with the SMDT mechanism proposed above.

It is also worth noting in Fig. 6 ▸ that, in general, the induction time and the transformation time of the SMDT process shortened significantly with the increase of *V*_w_ at the same temperature. The induction time and the transformation time of DMSO solvate at *V*_w_ = 0.225 were respectively 13.1 h and 41.2 h while they are 1.9 h and 6.7 h respectively when *V*_w_ = 0.275. This is because relatively high water activity can promote the formation of hydrate intermediates, resulting in a shorter induction time and transformation time. However, when *V*_w_ = 0.300, the induction time and the transformation time of the SMDT process were both anomalously longer than those of *V*_w_ = 0.275. This may be due to the strong hydrogen bond acceptor (HBA) propensity and hydrogen bond donor (HBD) propensity of water (Gu *et al.*, 2004 ▸), while LM have groups that have both HBA propensity and HBD propensity, such as its primary and secondary amide groups. Therefore, when water activity is higher, the tendency of forming a complex hydrogen bond network between LMs and water molecules enhances, resulting in more difficult removal of water molecules in the hydrate intermediate. Strong solute-solvent intermolecular interactions and low raw material concentration (because of the decreasing solubility of DMSO solvate as the water activity declined) will increase the difficulty of forming solute–solute intermolecular interactions, which will then increase the difficulty of nucleation of form D. As a result, the induction time and the transformation time will be prolonged. In fact, the reason why the viscosity of the system skyrockets when *V*_w_ > 0.300 might be also due to this mechanism.

### 3.5. Effect of temperature on the SMDT process   

**Figure 8:**
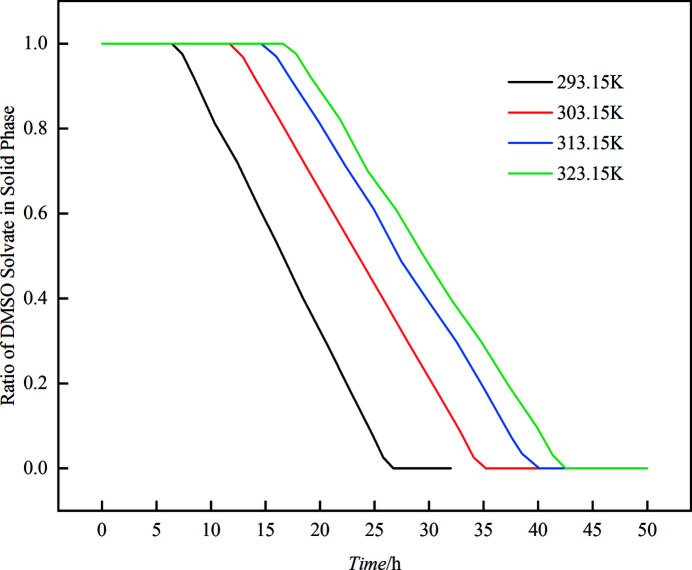
Effect of temperature on the SMDT process from LM DMSO solvate to form D in mixed solvent of DMSO and water (*V*_w_ = 0.250).

The effect of temperature on the desolvation process was also studied and the results are shown in Fig. 8 ▸. Obviously, the induction time and the transformation time of the SMDT process in the mixed solvent of *V*_w_ = 0.250 became longer as the temperature increased. The induction time increased from 6.4 h at 293.15 K to 16.6 h at 323.15 K, while the transformation time increased from 26.7 h at 293.15 K to 42.5 h at 323.15 K, as enumerated in Table 4 ▸.

There are two reasons for this abnormal result. Firstly, rising temperature will result in a lower thermodynamic driving force, which will lead to an increase in induction time and transformation time. Secondly, in the second step of the above four-step SMDT mechanism, the hydrogen bond between LMs and DMSO molecules in the solution would be broken while new hydrogen bond between LMs and water molecules will be formed. The higher temperature is not favorable for the formation of the new hydrogen bonds, which will affect the formation of crystal nuclei of form D. Thus, the transformation speed will gear down.

## 4. Conclusions   

Two new solid forms of LM were obtained and characterized by PXRD, TGA, DSC, PLM and Raman spectroscopy for the first time. To investigate the transformation behaviors from DMSO solvate to form D, the PXRD calibration curve was established to quantify the mass fraction of the two solid forms in a mixture. The solubility data of DMSO solvate and form D in DMSO–water mixed solvents were measured and correlated using NRTL equation. The solubility data were used to evaluate the thermodynamic driving force of the SMDT process from DMSO solvate to form D. It was found that a turning point at *V*_w_ = 0.225 existed. When *V*_w_ was relatively high, DMSO solvate would desolvate and transform to form D while the desolvation transformation process would not take place at lower *V*_w_. Through further investigations, it was found that the activity of water was the most important parameter that determined whether or not the SMDT process could happen. Moreover, Raman and solution concentration data displayed that the SMDT process was controlled by nucleation and growth of form D. Furthermore, the affecting mechanism of water activity and temperature on the SMDT process were investigated. The results demonstrated that the increase in temperature slowed down the SMDT process because of the decreasing thermodynamic driving force and the obstruction of forming new hydrogen bonds between LMs and water molecules. One new SMDT mechanism was suggested and discussed according to the experimental results and the mechanism was verified by cooling crystallization experiments.

## Supplementary Material

Figs S1-11 and Table S1. DOI: 10.1107/S2052520620003935/um5039sup1.pdf

## Figures and Tables

**Table 1:** All the discovered solid forms of LM Forms as reported by Matsushima *et al.* (2005 ▸) and Sardone *et al.* (2018 ▸).

Nomenclature	Solid form
Form A	Unsolvated form
Form B	Unsolvated form
Form C	Unsolvated form
Form F	Hydrate
Form I	Acetic acid solvate
Form DMSO-1	Hydrate
Form DMSO-2	Hydrate
Form ACA-1	Acetic acid solvate
Form ACA-1 HT dry	Unsolvated form
Form H_2_O-1	Unsolvated form
Form COF-1	Chloro­form solvate
Form FOA-1	Formic acid solvate

**Table 2:** The values of parameters in the equation for calculating molar solubility of LM

	ρ_d_ (g ml^−1^)	ρ_w_ (g ml^−1^)	*M*_d_ (g mol^−1^)	*M*_w_ (g mol^−1^)	*M*_LM_ (g mol^−1^)
293.15 K	1.100	0.998	78.13	18.02	601.09
303.15 K		0.996			(DMSO solvate)
313.15 K		0.992			522.96
323.15 K		0.988			(Form D)

**Table 3:** Induction time (*t*_ind_) and transformation time (*t*_tra_) of LM DMSO solvate to form D in DMSO–water mixed solvent with different *V*_w_

	*V*_w_			
	0.225	0.250	0.275	0.300
*t*_ind_ (h)	13.1	6.4	1.9	2.5
*t*_tra_ (h)	41.2	26.7	6.7	8.2

**Table 4:** Induction time and transformation time of LM DMSO solvate to form D at different temperatures

	*T* (K)			
	293.15	303.15	313.15	323.15
*t*_ind_ (h)	6.4	11.7	14.8	16.6
*t*_tra_ (h)	26.7	35.2	40.1	42.5
