# Supplementary material for: The mechanism of solvent-mediated desolvation transformation of lenvatinib mesylate from di­methyl sulfoxide solvate to form D
Source: Acta Crystallogr B Struct Sci Cryst Eng Mater. 2020 May 7;76(Pt 3):343–52. doi: 10.1107/S2052520620003935 (PMC7278088; doi:10.1107/S2052520620003935)
Supplement: Supplementary file 1 [file b-76-00343-sup1.pdf]

Acta Cryst B

STRUCTURAL SCIENCE
CRYSTAL ENGINEERING
MATERIALS

**Volume 76 (2020)**

**Supporting information for article:**

# The mechanism of solvent-mediated desolvation transformation of lenvatinib mesylate from dimethyl sulfoxide solvate to form D

**Zhixin Zheng, Baohong Hou, Xiaowei Cheng, Wanying Liu, Xin Huang, Ying Bao, Ting Wang, Zhao Wang and Hongxun Hao**

## 1 Characterization data of DMSO Solvate and Form D of LM

The characterization data of PXRD, PLM, TGA, DSC and Raman spectroscopy of LM DMSO solvate and Form D are shown in Figure S1 to Figure S6:

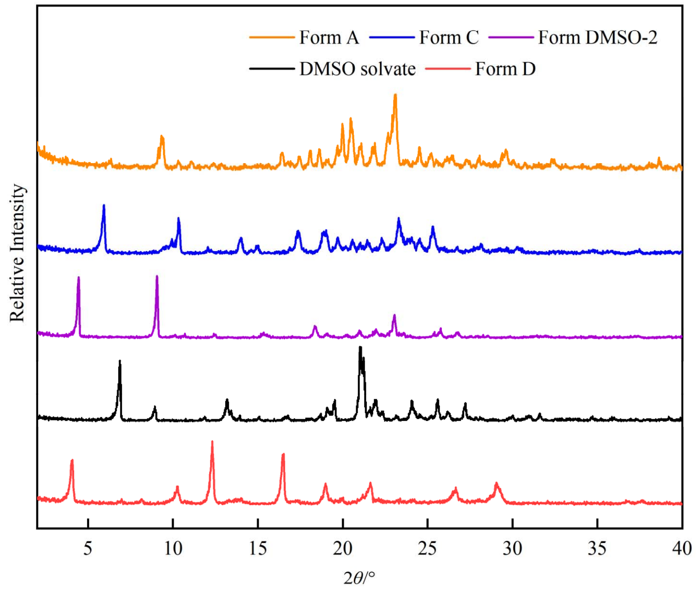


Figure S1. PXRD patterns of Form A (reported), Form C (reported), Form DMSO-2 (reported), DMSO solvate and Form D of LM.

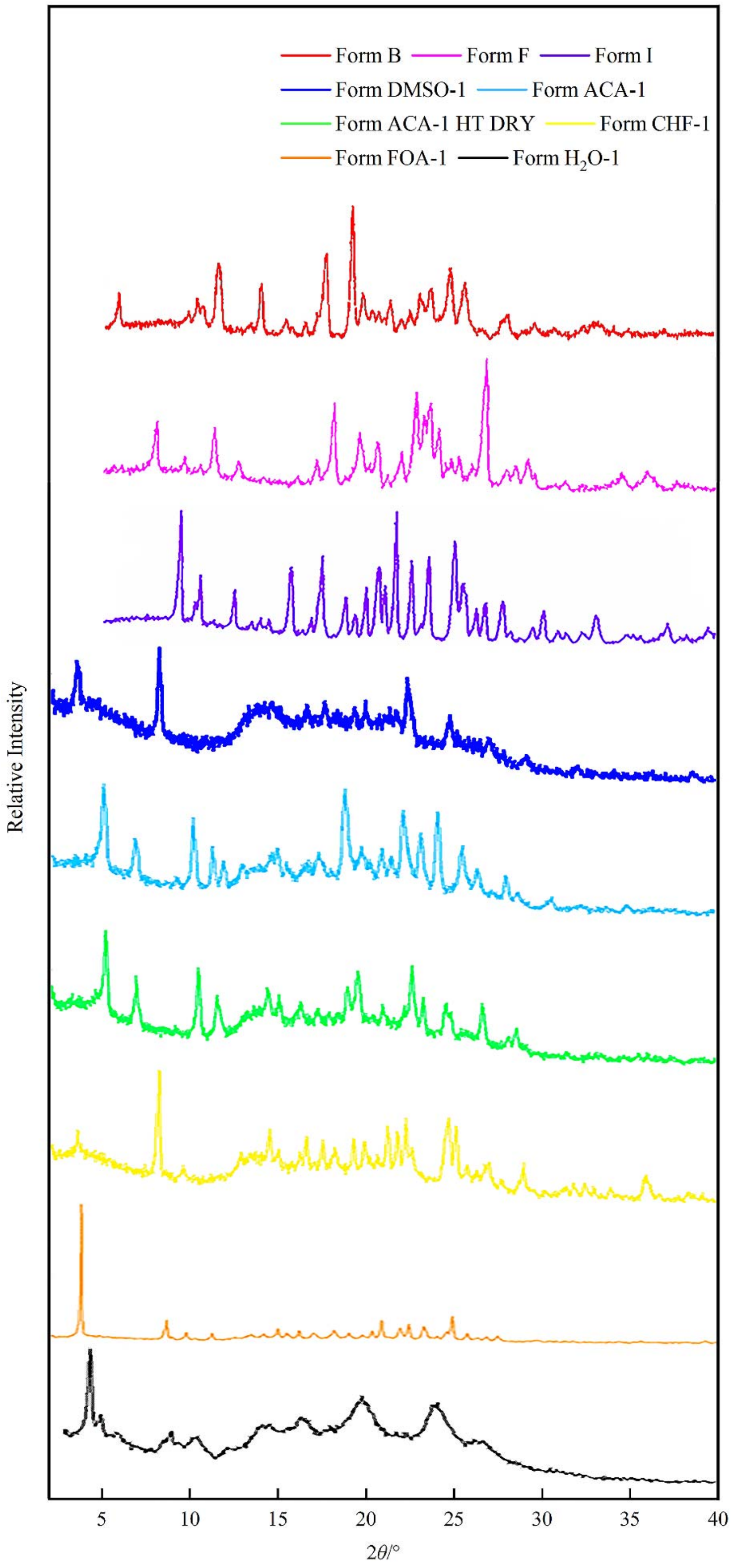


Figure S2. PXRD patterns of Form B (reported), Form F (reported), Form I (reported), Form DMSO-1 (reported), Form ACA-1 (reported), Form ACA-1 HT

DRY (reported), Form CHF-1 (reported), Form FOA-1 (reported) and Form $H_2O$-1 (reported) of LM.

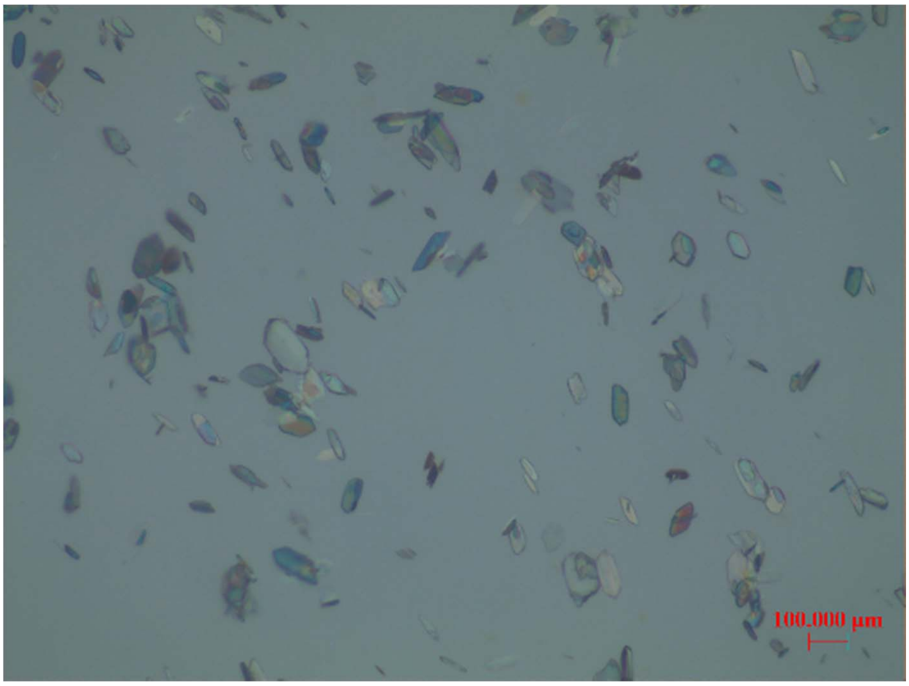


(a)

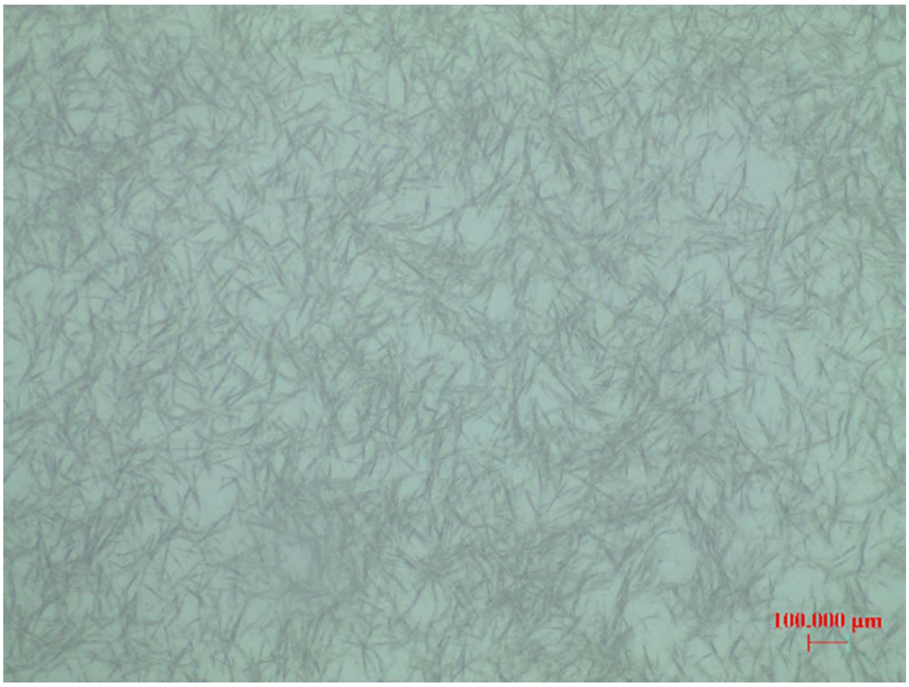


(b)

Figure S3. The crystal morphology of DMSO solvate and Form D of LM. (a) DMSO solvate, and (b) Form D.

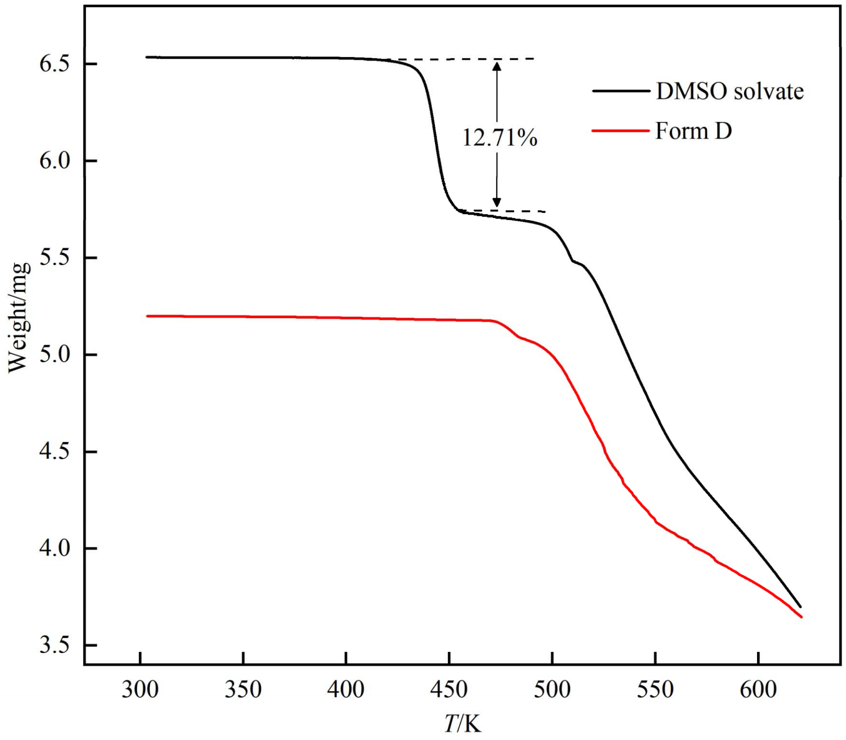


Figure S4. TGA thermograms of DMSO solvate and Form D of LM.

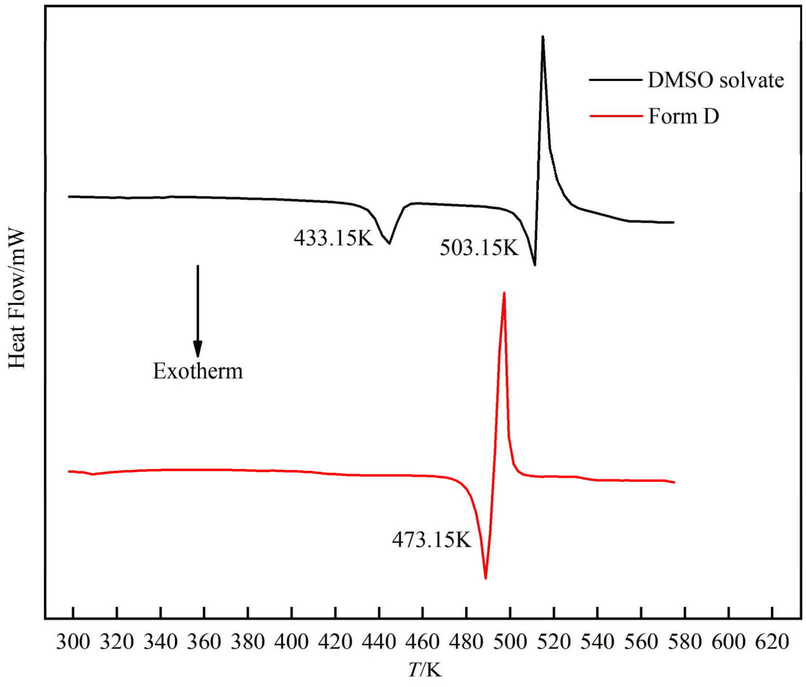

Figure S5. DSC curves of DMSO solvate and Form D of LM.

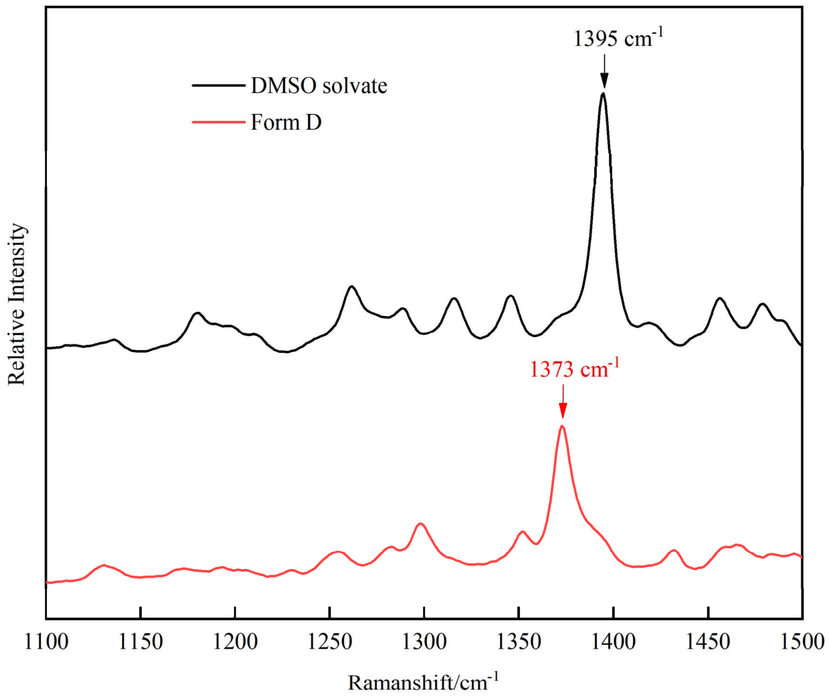


Figure S6. Raman spectra (part) of DMSO solvate and Form D of LM.

## 2 Calibration Curve for Quantitative Analysis Method

$2\theta$= 6.9 ± 0.2 ° and $2\theta$= 12.3 ± 0.2 ° were respectively selected as the characteristic peaks of DMSO solvate and Form D to establish a quantitative calibration curve. The relative characteristic peak intensity of DMSO solvate was defined as $x$ axis while the actual mass fraction of DMSO solvate was defined as $y$ axis. The results are shown in Figure S7. The value of $R^2$ of the calibration curve was fitted as 0.9956, which indicates that the calibration curve has a good linearity. Based on the characteristic peak intensities of DMSO solvate and Form D in the PXRD diffraction patterns of the mixture, the relative mass fraction of DMSO solvate could be calculated by this quantitative calibration curve.

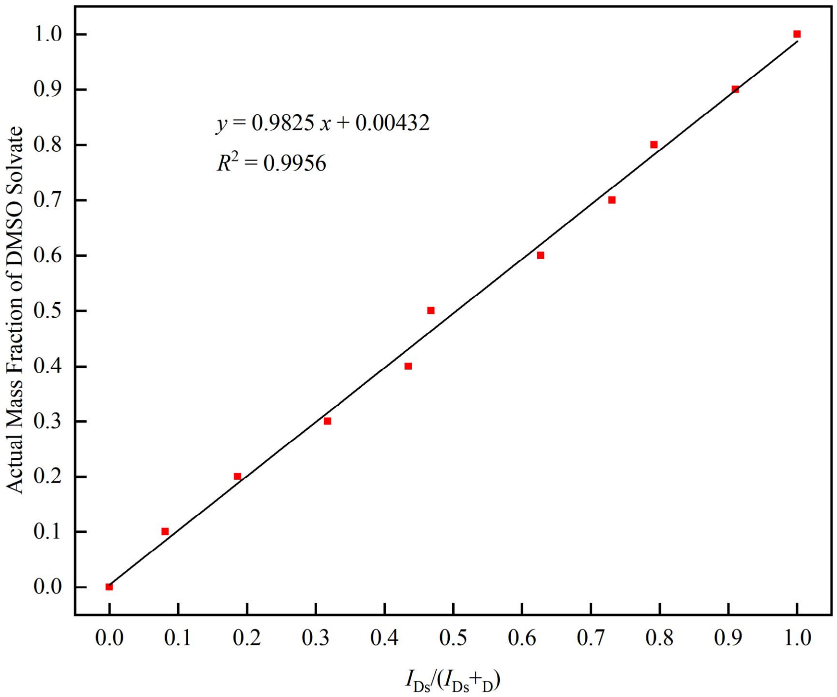


Figure S7. The PXRD calibration curve of DMSO solvate and Form D of LM.

## 3 Thermodynamic Data of DMSO Solvate and Form D of LM in Solution Mixtures of DMSO and Water

The solubility data of LM DMSO solvate and Form D in the mixed solvents of DMSO and water were measured. Furthermore, the activities of DMSO solvate/Form D, water and DMSO in saturated solution systems were calculated by NRTL equation, and all results are listed in Table S1.

Table S1. Thermodynamic data of DMSO solvate and Form D of LM in solution mixtures of DMSO and water[a].

| $V_w$ | DMSO solvate | | Form D | | $a_w$ | $a_d$ | $a_{Ds}/10^{-3}$ | $a_D/10^{-4}$ |
|---|---|---|---|---|---|---|---|---|
| | $x_{exp}/10^{-3}$ | $x_{cal}/10^{-3}$ | $x_{exp}/10^{-3}$ | $x_{cal}/10^{-3}$ | | | | |
| $T$= 293.15 K | | | | | | | | |
| 0 | 4.295 | 4.446 | 2.695 | 2.804 | 0 | 0.9967 | 1.012 | 0.6642 |
| 0.025 | 4.018 | 4.166 | 2.167 | 2.212 | 0.05127 | 0.9034 | 1.010 | 0.6771 |
| 0.050 | 3.774 | 3.917 | 1.734 | 1.768 | 0.1003 | 0.8185 | 1.009 | 0.6777 |
| 0.075 | 3.564 | 3.698 | 1.377 | 1.401 | 0.1472 | 0.7412 | 1.010 | 0.6796 |
| 0.100 | 3.376 | 3.501 | 1.084 | 1.099 | 0.1924 | 0.6706 | 1.010 | 0.6817 |
| 0.125 | 3.207 | 3.325 | 0.8436 | 0.8542 | 0.2358 | 0.6062 | 1.011 | 0.6824 |
| 0.150 | 3.056 | 3.167 | 0.6471 | 0.6578 | 0.2777 | 0.5473 | 1.011 | 0.6799 |
| 0.175 | 2.919 | 3.025 | 0.4879 | 0.5017 | 0.3181 | 0.4936 | 1.011 | 0.6721 |
| 0.200 | 2.794 | 2.896 | 0.3603 | 0.3790 | 0.3571 | 0.4446 | 1.011 | 0.6570 |
| 0.225 | 2.681 | 2.780 | 0.2596 | 0.2835 | 0.3941 | 0.3859 | 1.010 | 0.6329 |
| 0.250 | 2.577 | 2.675 | 0.1820 | 0.2099 | 0.4325 | 0.3445 | 1.009 | 0.5993 |
| 0.275 | 2.481 | 2.580 | 0.1244 | 0.1539 | 0.4697 | 0.3070 | 1.008 | 0.5585 |
| 0.300 | 2.393 | 2.493 | 0.0900 | 0.1116 | 0.5055 | 0.2733 | 1.006 | 0.5576 |
| $T$= 303.15 K | | | | | | | | |
| 0 | 5.592 | 5.448 | 2.920 | 2.816 | 0 | 0.9959 | 1.850 | 1.644 |
| 0.025 | 5.231 | 5.102 | 2.354 | 2.279 | 0.05290 | 0.9027 | 1.847 | 1.638 |

| | | | | | | | | |
|---|---|---|---|---|---|---|---|---|
| 0.050 | 4.916 | 4.796 | 1.889 | 1.841 | 0.1033 | 0.8181 | 1.847 | 1.628 |
| 0.075 | 4.638 | 4.523 | 1.507 | 1.469 | 0.1515 | 0.7410 | 1.847 | 1.626 |
| 0.100 | 4.391 | 4.281 | 1.192 | 1.161 | 0.1977 | 0.6708 | 1.848 | 1.629 |
| 0.125 | 4.171 | 4.063 | 0.9341 | 0.9089 | 0.2421 | 0.6067 | 1.850 | 1.630 |
| 0.150 | 3.973 | 3.868 | 0.7231 | 0.7051 | 0.2847 | 0.5483 | 1.850 | 1.626 |
| 0.175 | 3.794 | 3.693 | 0.5518 | 0.5421 | 0.3257 | 0.4950 | 1.851 | 1.614 |
| 0.200 | 3.631 | 3.535 | 0.4142 | 0.4130 | 0.3650 | 0.4464 | 1.851 | 1.591 |
| 0.225 | 3.482 | 3.391 | 0.3055 | 0.3119 | 0.4050 | 0.3889 | 1.850 | 1.553 |
| 0.250 | 3.346 | 3.262 | 0.2214 | 0.2334 | 0.4437 | 0.3479 | 1.849 | 1.504 |
| 0.275 | 3.221 | 3.144 | 0.1585 | 0.1730 | 0.4809 | 0.3108 | 1.846 | 1.452 |
| 0.300 | 3.106 | 3.036 | 0.1215 | 0.1270 | 0.5167 | 0.2774 | 1.843 | 1.518 |

$T$= 313.15 K

| | | | | | | | | |
|---|---|---|---|---|---|---|---|---|
| 0 | 6.915 | 6.741 | 3.185 | 3.073 | 0 | 0.9951 | 3.071 | 3.577 |
| 0.025 | 6.354 | 6.250 | 2.576 | 2.512 | 0.05444 | 0.9021 | 3.044 | 3.540 |
| 0.050 | 5.970 | 5.872 | 2.076 | 2.041 | 0.1062 | 0.8177 | 3.044 | 3.511 |
| 0.075 | 5.633 | 5.537 | 1.665 | 1.639 | 0.1556 | 0.7409 | 3.045 | 3.506 |
| 0.100 | 5.332 | 5.238 | 1.326 | 1.303 | 0.2027 | 0.6710 | 3.047 | 3.512 |
| 0.125 | 5.064 | 4.971 | 1.047 | 1.026 | 0.2479 | 0.6073 | 3.050 | 3.521 |
| 0.150 | 4.822 | 4.731 | 0.8190 | 0.8016 | 0.2912 | 0.5492 | 3.052 | 3.526 |
| 0.175 | 4.605 | 4.515 | 0.6337 | 0.6209 | 0.3326 | 0.4963 | 3.053 | 3.522 |
| 0.200 | 4.407 | 4.320 | 0.4845 | 0.4771 | 0.3724 | 0.4481 | 3.053 | 3.505 |

| | | | | | | | | |
|---|---|---|---|---|---|---|---|---|
| 0.225 | 4.225 | 4.144 | 0.3662 | 0.3635 | 0.4151 | 0.3918 | 3.052 | 3.477 |
| 0.250 | 4.060 | 3.984 | 0.2743 | 0.2747 | 0.4540 | 0.3512 | 3.051 | 3.447 |
| 0.275 | 3.907 | 3.838 | 0.2048 | 0.2059 | 0.4912 | 0.3145 | 3.047 | 3.433 |
| 0.300 | 3.767 | 3.706 | 0.1586 | 0.1529 | 0.5269 | 0.2814 | 3.043 | 3.578 |
| *T*= 323.15 K | | | | | | | | |
| 0 | 8.308 | 8.425 | 3.524 | 3.615 | 0 | 0.9943 | 4.753 | 6.978 |
| 0.025 | 7.767 | 7.882 | 2.863 | 2.934 | 0.05590 | 0.9014 | 4.750 | 6.985 |
| 0.050 | 7.296 | 7.402 | 2.319 | 2.384 | 0.1089 | 0.8171 | 4.751 | 6.965 |
| 0.075 | 6.880 | 6.976 | 1.872 | 1.919 | 0.1593 | 0.7407 | 4.753 | 6.980 |
| 0.100 | 6.511 | 6.597 | 1.503 | 1.532 | 0.2074 | 0.6711 | 4.757 | 7.020 |
| 0.125 | 6.182 | 6.258 | 1.199 | 1.214 | 0.2533 | 0.6078 | 4.761 | 7.072 |
| 0.150 | 5.885 | 5.954 | 0.9499 | 0.9538 | 0.2972 | 0.5501 | 4.764 | 7.129 |
| 0.175 | 5.617 | 5.680 | 0.7470 | 0.7440 | 0.3391 | 0.4975 | 4.766 | 7.187 |
| 0.200 | 5.373 | 5.433 | 0.5832 | 0.5760 | 0.3791 | 0.4497 | 4.767 | 7.248 |
| 0.225 | 5.151 | 5.209 | 0.4528 | 0.4427 | 0.4244 | 0.3945 | 4.766 | 7.322 |
| 0.250 | 4.947 | 5.005 | 0.3510 | 0.3377 | 0.4634 | 0.3543 | 4.763 | 7.441 |
| 0.275 | 4.760 | 4.821 | 0.2692 | 0.2557 | 0.5007 | 0.3180 | 4.759 | 7.537 |
| 0.300 | 4.586 | 4.652 | 0.2102 | 0.1922 | 0.5362 | 0.2853 | 4.752 | 7.830 |

[a] $x_{exp}$ and $x_{cal}$ represent the experimental measurement values of solubility and the calculated values of solubility fitted by NRTL equation, respectively. $a_{Ds}$, $a_d$, $a_w$ and

$a_D$ represent the activity of DMSO solvate, Form D, water and DMSO in saturated solution of LM, respectively.

**4 PXRD Patterns of Solid Phase at Different Moments in Rate Control Step Determination Experiment**

PXRD patterns of suspended solid in rate control step determination experiments are shown in Figure S8.

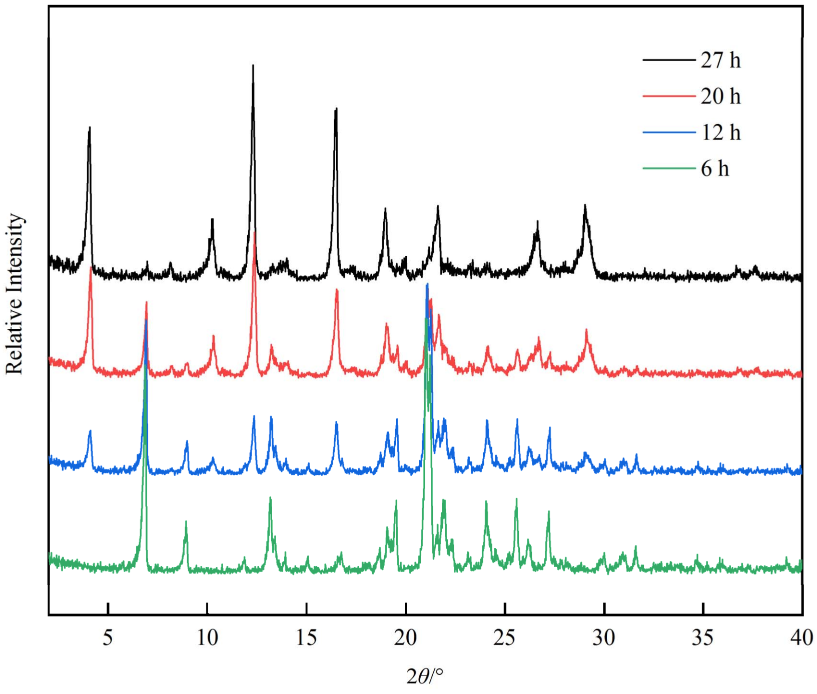


Figure S8. PXRD patterns of solid phase at different moments.

**5 Characterization of Form DMSO-2 of LM**

The PXRD, TGA, and DSC characterization results of Form DMSO-2 of lenvatinib mesylate are shown in Figure S9, S10 and S11.

DMSO-2 of lenvatinib mesylate has particular characteristic peaks at $2\theta$= 4.4, 9.0, 15.4, 18.3, 20.9, 23.0, 25.7, 26.8 ± 0.2 °, as shown in Figure S9.

It can be seen from Figure S10 and S11 that Form DMSO-2 of lenvatinib mesylate has a weight loss of 12.07% at about 379.25 K, which is corresponding to the endothermic peak at the same temperature in DSC curve. The weight loss is consistent with the theoretical water content in a lenvatinib mesylate tetrahydrate (about 12.11%), which confirms that Form DMSO-2 is a tetrahydrate of lenvatinib mesylate. Another endothermic peak at 498.85 K indicates that crystals formed after desolvation of Form DMSO-2 melts around 498.85 K.

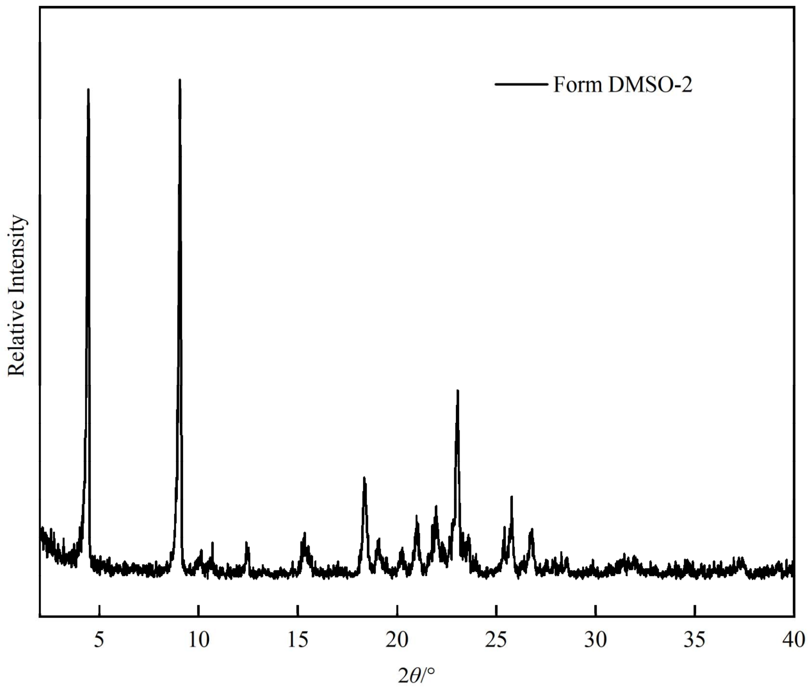


Figure S9. PXRD pattern of Form DMSO-2 of lenvatinib mesylate.

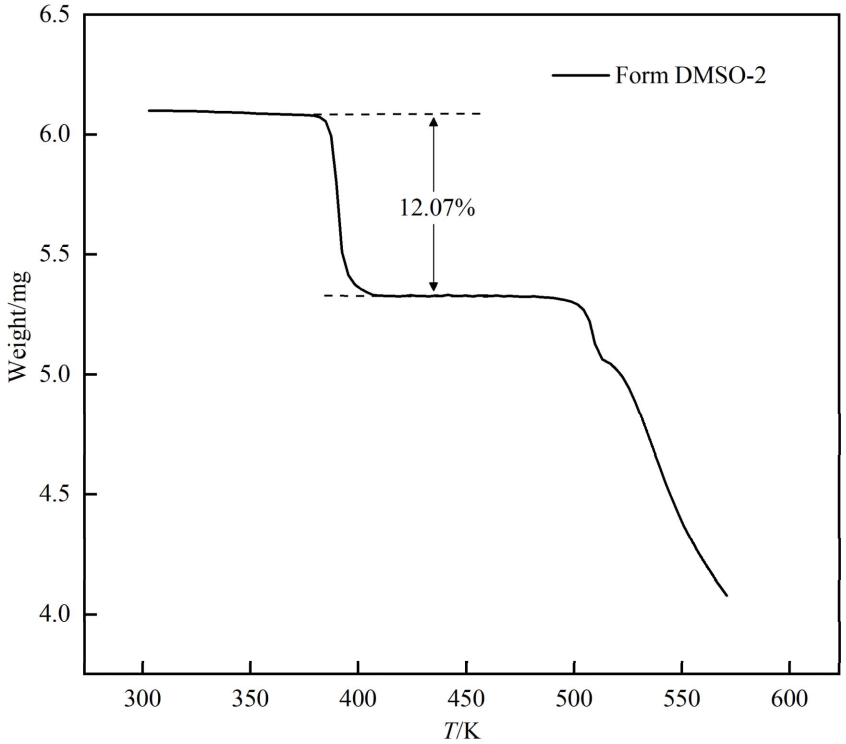


Figure S10. TGA thermogram of Form DMSO-2 of lenvatinib mesylate.

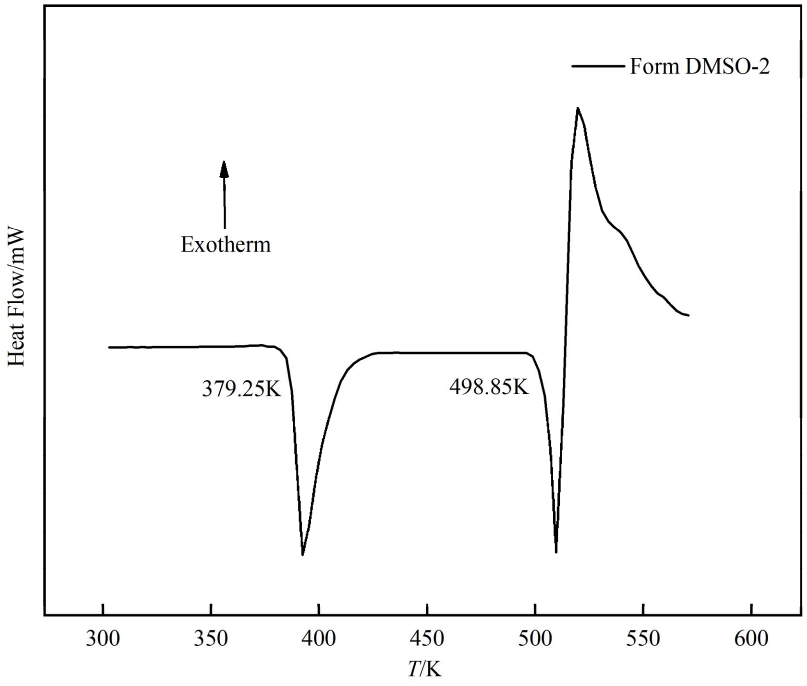


Figure S11. DSC curve of Form DMSO-2 of lenvatinib mesylate.
